# Microglial Exosomes in Neurodegenerative Disease

**DOI:** 10.3389/fnmol.2021.630808

**Published:** 2021-05-11

**Authors:** Min Guo, Yining Hao, Yiwei Feng, Haiqing Li, Yiting Mao, Qiang Dong, Mei Cui

**Affiliations:** ^1^Department of Neurology, Huashan Hospital, Fudan University, Shanghai, China; ^2^Department of Radiology, Huashan Hospital, Fudan University, Shanghai, China; ^3^Department of Neurology, Huashan Hospital, State Key Laboratory of Medical Neurobiology and MOE Frontiers Center for Brain Science, Fudan University, Shanghai, China

**Keywords:** microglia, exosome, neurodegenerative disease, Alzheimer’s disease, Parkinson’s disease, amyotrophic lateral sclerosis

## Abstract

Microglia play an important role in neurodegenerative disease [i.e., Parkinson’s disease (PD), Alzheimer’s disease (AD), and amyotrophic lateral sclerosis (ALS)]. These diseases share some similar pathological changes and several microglia-associated processes, including immune response, neuroinflammation, phagocytosis, elimination of synapses et al. Microglia in the central nervous system (CNS) has been described as having both destructive and protective effects in neurological disorders. Besides, considerable evidence also indicates that microglia play a significant role in neurogenesis, neuronal cell death, and synaptic interactions. The communication between microglia and neurons is of vital role in regulating complex functions which are key to appropriate the activity of the brain. Accumulating studies have also demonstrated that exosomes with sizes ranging from 40–100 nm, released by microglia, could serve as key mediators in intercellular signaling. These exosomes, identified in terms of cellular origin in many kinds of biological fluids, exert their effects by delivering specific cargos such as proteins, microRNAs (miRNAs), and mRNAs. It was shown that microglial exosomes could transport to and be uptake by neurons, which may either be beneficial or instead, detrimental to CNS diseases. The focus of this review is to summarize the involvement of microglial exosomes in critical pathologies associated with neurodegenerative disease and how they contribute to these disorders, including PD, AD, and ALS. We also review the application of microglia exosomes as potential biomarkers in monitoring disease progression, as well as focusing on their roles as drug delivery vehicles in treating neurodegenerative disorders.

## Background

The communication between diverse cellular populations is a characteristic of neurodegenerative disorders, with different ways including exocytosis, exosomes, tunneling nanotubes, and endocytosis (Fruhbeis et al., 2013; Peferoen et al., 2014; Valdinocci et al., 2017). Neurons, the effector cells in the brain, have interaction with microglia, astrocytes, oligodendrocytes, as well as the neurovascular system, supporting their metabolic requests and respond to pathological stimuli. Conversely, this kind of communication can also promote the progression of the neurodegenerative disease through spreading harmful agents such as cytokines and pathogenic proteins. One emerging model of propagating such cell-to-cell interplay is through the release of disease-related exosomes in neurodegenerative disorders (Rufino-Ramos et al., 2017; Yuyama and Igarashi, 2017). Once released by cells in the brain, exosomes can either target specific cell types or be released into the cerebrospinal fluid (CSF) and blood (Basso and Bonetto, 2016), as well as uptaken by neighboring cells. Indeed, exosomes were detected in the CSF of humans as well as model species such as mice and monkeys and cell culture medium (Basso and Bonetto, 2016), thus leading to the possibility that exosomes may be useful for early diagnosis in neurodegenerative diseases.

## Microglia in Neurodegenerative Diseases

As the principal immune-competent cells in the CNS, microglia play an active role in influencing the pathologic progression of AD, ALS, and PD, accompanied by altering their morphology, transcriptional profile, and functions when become activated (Brites and Vaz, 2014). Under homeostatic conditions, microglia exist in a resting state with thin processes spanning the brain where they survey for pathological or harmful proteins/molecules such as α-synuclein, tau, or Aβ, which may be free in the extracellular matrix or included in exosomes. Detection of these stimulating agents causes the motile microglia to change from a relative resting state to a phagocytic state, with a morphological transformation from a ramified form to an amoebic form (Gemma and Bachstetter, 2013). Activated microglia exert phagocytic capacity on free and exosome associated misfolding proteins or other harmful agents from neurons, astrocytes, or oligodendrocytes, exerting protective effects. In parallel, microglia are also capable of sensing molecular or cellular debris and then phagocyte and degrade them. Activated microglia exert different functions in different mouse disease models and humans. Microglia acquire alternate phenotypes, such as the M1 (inflammatory category) and M2 subtypes (pro-regenerative category), depending on the differing stimuli they are to respond to and the extent of the stimuli (Brites and Vaz, 2014). While distinguishing the function of microglia as either “protective” or “injury” is relatively difficult, because microglia are dynamically switching between these phenotypes and may exist in either M1, M2 or, intermediate states. Recently, the “disease-associated microglia” (DAM) is discovered in AD by comprehensive single-cell RNA analysis and later investigations also discovered DAM in ALS (Keren-Shaul et al., 2017; Olah et al., 2018). DAM refers to a subset of microglia with a unique transcriptional and functional feature, expressing typical microglial markers, such as Iba1 and Hexb, together with elevation of genes involved in lysosomal and phagocytic pathways, such as Apoe, Ctsd, and Trem2 (Lambert et al., 2013). DAM can be activated by various stimuli, including protein aggregates, myelin debris and cell debris, depending on TREM2–DAP12 pathway, and can participate in the clearance of apoptotic cells, myelin debris, Aβ and secrete inflammatory cytokines (Deczkowska et al., 2018). The focus of microglia is gradually shifted from the M1 and M2 paradigm to DAM associated with neurodegeneration.

## Characteristics of Microglial Exosomes

Intracellularly, microRNAs (miRNAs), messenger RNAs (mRNAs), proteins, and cytokines can be selectively incorporated into multivesicular bodies (MVBs) and then secreted from microglia encapsulated in exosomes. Exosomes are small vesicles (40–100 nm) generated from the intracellular endosomal system. The inward budding of the endocytic membrane forms MVBs and exosomes are secreted through exocytosis when MVBs fuse with plasma membranes (Prada et al., 2013). There are another set of extracellular vesicles from microglia called microvesicles (MV), which are relatively large vesicles (100–1,000 nm) that release from the directly budding of the plasma membrane (Turola et al., 2012). Comparisons between these two types of vesicles have found that exosomes are primarily enriched with receptors and kinases, indicating their function in cellular signaling. Whereas MVs are presented with mitochondrial, centrosomal, and ribosomal proteins, indicating their involvements in protein translation (Basso and Bonetto, 2016). In addition, another important difference is that microglial exosomes have been identified as having a unique role in antigen presentation, for instance, the transfer of antigens, implying that exosomes exert a functional role in immune response and are vital in managing the interaction between the brain and the immune system. To sum up, due to the unique features of exosomes in intercellular communications acting as vehicles, and promoting disease propagation as essential carriers of altered molecules, their functional roles in neurodegenerative disease are attracting more attention.

Exosomes are generated from the late endosome to form MVBs, where ESCRT protein complexes coordinate the cargo loading and vesicle release (Baietti et al., 2012). Although the mechanisms underlying the fusion of MVBs with the plasma membrane is still poorly understood, several Rab GTPases, such as the SNARE protein YKY6, and Rab11, 35, 27 have been shown to coordinate vesicle tethering and the fusion of MVBs to the plasma membrane (Chevallier et al., 2009; Gross et al., 2012; Shifrin et al., 2013; Binotti et al., 2016). The cargos of exosomes are known to comprise proteins, lipids, and RNAs, while the presence of DNA is highly contested. Exosomes themselves are lined by bi-lipid bilayers enriched in certain membrane proteins such as tetraspanins, cholesterol, and sphingomyelin. Exosomes have been found to enter recipient cells through endocytosis, ligand-receptor interaction (although identification of specific ligands and receptors has not yet been made), or fusion with the plasma membrane (Ratajczak et al., 2006).

Several factors have been proven to influence the release of exosomes from microglia. Microglial exosome secretion can be restrained with GW4869, an inhibitor of sphingomyelin-2 that targets neutral sphingomyelinase-2 (Yuyama et al., 2012). Upon ATP stimulation of P2X7 receptors, reactive microglia release exosomes rapidly (Gu et al., 2011). 5-HT can promote the release of microglial exosomes, which is mediated by cAMP-GEF1/2 in 5-HT_4_R signaling and involves the elevation of (Ca^2+^) levels (Glebov et al., 2015). It also has been reported that the release of microglial exosomes does not occur constitutively and the release is induced by Wnt3a, a cysteine-rich glycoprotein, which in turn be included in exosomes (Hooper et al., 2012).

**FIGURE 1:**
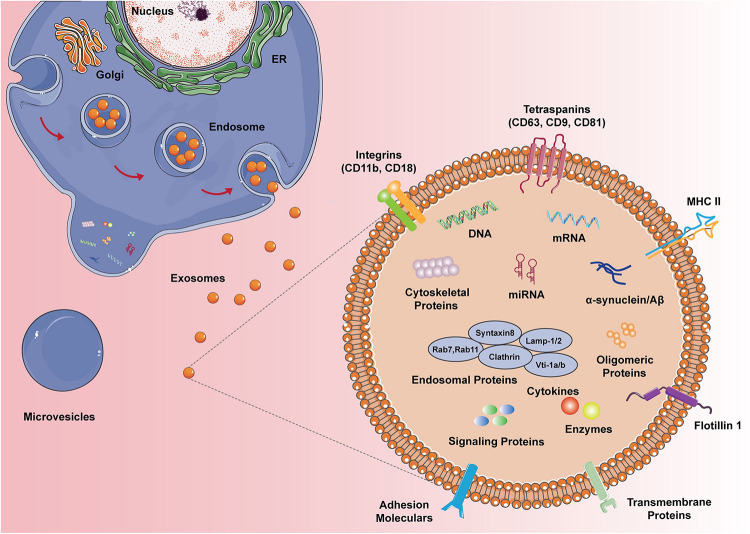
The composition of microglial exosomes. Different from the microvesicles that bus outward from the plasma membrane, exosomes are generated from the endocytic pathway and secretory pathway, hence exosomes carry multiple proteins, DNA, RNA, and lipids from the original cells. Microglial exosomes express endosomal proteins, cytoskeletal proteins, enzymes, and specific MHC II and CD11b. Depending on the status of microglia, exosomes can also have cytokines, signaling proteins, and disease proteins like α-syn and Aβ.

Based on the proteomic analysis of exosomes from N9 microglial cells (Potolicchio et al., 2005), the composition of typical microglial exosomes is illustrated in Figure 1. Microglia-derived exosomes contain proteins of the late endosomes and these proteins serve as specific markers of exosomes corroborating their organelle origin. Microglial exosomes also express major histocompatibility complexes (MHCs) class II molecules, yet the co-stimulatory molecules like CD80, CD86 are not found in the bilayers near MHC II, thus their roles in antigen presentation andtolerance inductionin the brain isremainedto be proven (Thomas et al., 2007). Tetraspanins are a family of transmembrane proteins that are integrated into the membrane of exosomes. CD9, CD81, and CD63 are tetraspanins that are particularly enriched in microglial exosomes and are usually used as exosomal markers. Flotillin 1, another commonly used exosomal marker, is a membrane-associated protein that is highly expressed in microglial exosomes. The monocyte/macrophage marker CD14 is also expressed in microglial exosomes and is regularly used to characterize exosomal preparations. The lactate transporter MCT-1 and the unique surface-bound aminopeptidase CD13 are also enriched in exosomes derived from microglia, which may participate in the energy substrate supply to neurons during synaptic activity. Interestingly, microglia-specific protein CD11 is also contained in exosomes. Extrusion of Na, K-ATPase is similarly frequent in microglial exosomes. Exosomes also contain the pro-inflammatory cytokines (e.g., TNF-α, IL-1b) and release them to extracellular space when MVB fused with the plasma membrane of the microglia upon ATP stimulation. Besides, exosomes carry a distinct group of proteins that are expressed as housekeeping proteins, such as GAPDH. Nevertheless, taking into consideration that the transcriptomic characterization of the N9 immortalized microglia cell line does not exactly line up with primary microglia, further studies are sufficiently needed to depict the proteomic composition of primary microglia-derived exosomes.

**FIGURE 2:**
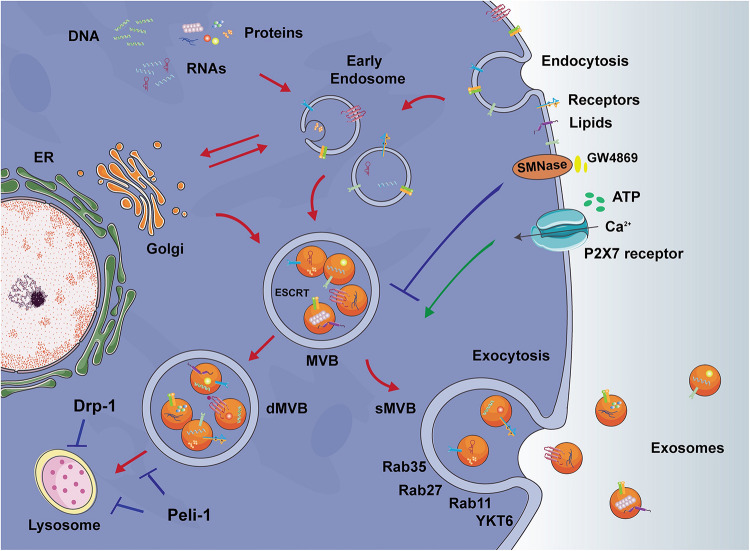
Exosome formation and release. Endocytic pathway: Early endosomes are vesicles that originate by endocytosis at the plasma membrane. The membrane of the endosome then bud inwardly into the lumen of the endosome and form multivesicular bodies (MVBs). Secretory MVBs (sMVBs) are the cellular source of exosomes. sMVBs then fuse with the plasma membrane, releasing exosomes into the extracellular space. Secretory pathway: In the process of endosome and MVBs formation, the Golgi complex fuses with the membrane of endosome or MVBs and thus leading to the mixed membrane constituents of exosomes, including proteins from the plasma membrane or Golgi complex. Degradative MVBs (dMVBs) subsequently fuse with the lysosome, leading to MVBs content degradation, thus lysosome dysfunction directly influences the exosome release. Inhibiting E3 ubiquitin ligase Peli-1 or mitochondria fission protein Drp-1 in activated microglia might help to improve lysosome function and restore autophagy flux, hence regulating exosomes release. Other key targets to regulate exosome release include sphingomyelinase-2 (SMNase) and P2X7 receptors. SMNase is the receptor of small molecular GW4869 and the activation of which inhibits exosome release. P2X7 receptors can bind with ATP and promote the exosome release.

Studies have revealed that microglial exosomes carry the proteins related to neurodegenerative diseases. It was reported that activated microglia has a strong ability to release exosomes, containing misfolded proteins like α-synuclein, tau, Aβ (Kosaka et al., 2010; Shi et al., 2014; Asai et al., 2015; Sardar Sinha et al., 2018; Guo et al., 2020), and cytokines (Fernandes et al., 2018). They also carry the insulin-degrading enzyme (IDE), which can also degrade the Aβ peptide (Kosaka et al., 2010). An important feature of microglial cells is their high plasticity upon activation, allowing them to release exosomes which can spread to distal brain regions. Thus, activated microglia may mediate the spread of misfolded proteins either *via* exosomes or *via* membrane leakage after the death of the migrated cells, making microglia an effective transporter during disease (Turola et al., 2012). The connection of microglial exosomes to the pathogenesis and development of AD has raised a great deal of attention.

## Microglia and Their Exosomes in AD

Alzheimer’s disease (AD) has been clinically characterized by progressive dementia with pathological deposition of neurofibrillary tangles (NFTs) and β-amyloid (Aβ) plaque. Decades before the occurrence of cognitive dysfunction, the hyperphosphorylated tau, combined with excessive aggregation of Aβ, have been identified as two of the most important hallmarks in AD brains. An array of studies have suggested the intricate and controversial role of exosomes in AD. And the exosomal communication between microglia and other cells has determined them as an appealing target for developing therapeutic approaches.

Exosome derived from the CNS has been found in many kinds of bodily fluids, including the cerebrospinal fluids (CSF). Despite the presence of tau trimers and monomers that have been identified in exosomes isolated from the CSF of both AD patients and control individuals, no difference has been observed between the two groups (Wang et al., 2017). In contrast, *in vitro* experiment has demonstrated that exosomes from hTau treated primary cultured murine microglia contained tau (Asai et al., 2015). In line with this, Asai et al. (2015) have revealed in their recent finding that tau is able to transfer from microglia to neurons through the exosome-synaptic pathway, as blocking the synthesis of exosomes or depleting microglia in mice mitigated the deposition of tau in the surrounding healthy cells. Furthermore, microglia-derived tau-containing exosomes were able to be taken up by neurons and triggered abnormal aggregation of tau, thus indicating a synergistic role between microglia and exosome in the pathological spread of tau (Asai et al., 2015).

Besides the mentioned roles, several lines of evidence are also available showing that inhibiting the secretion of exosomes in the 5XFA model of AD is capable of decreasing the amyloid plaque load in brains (Dinkins et al., 2014, 2015). It has also been proved that exosome-derived proteins are detectable in Aβ plaques of AD patients (Dinkins et al., 2014), indicating that exosomes act as potential carriers of the pathological proteins in deteriorating AD pathology. In addition, presenilin, APP, C-terminal fragments of APP (APP-CTFs), and several key proteases in Aβ production such as β-secretase (BACE1) and γ-secretase (presenilin subunits PS1 or PS2) are also found in exosomes isolated from AD brain tissues (Perez-Gonzalez et al., 2012). A recent study conducted by Sardar Sinha et al. (2018) revealed that exosomes could carry oligomeric Aβ and mediate the Aβ transmission to neighboring neurons, illustrating an exosome-mediated propagation of AD toxic species. In line with this, there are also studies demonstrating that MVs from microglia pre-exposed with 4 mM Aβ contain soluble Aβ (Shi et al., 2014), and primary microglia have a strong ability to internalize Aβ protofibrils and release MVs, which contain the transmissible Aβ peptides (Gouwens et al., 2018). Moreover, Joshi et al. (2014) have shown that microglia derived MVs could strongly increase Aβ neurotoxicity, which is due to the promotion of Aβ1–42 extracellular aggregates to form small soluble neurotoxic species by lipid components of MVs.

In addition to the above fact that microglial exosomes are able to carry tau and Aβ, evidence also showed that microglial exosomes act as negative regulators in AD pathogenesis in multiple ways. One possible aspect is that microglia-derived exosomes deliver proinflammatory signals. Verderio et al. (2012) have revealed in their study that MVs (mixed with exosomes) from LPS pre-activated microglia caused a dose-dependent activation of astrocytes and microglia. Besides, a recent study conducted by Fernandes has also reported that microglia were able to internalize exosomes released from APP-overexpressed SH-SY5Y cells, which subsequently induced microglia activation and pro-inflammatory cytokines release (Fernandes et al., 2018).

Neuron-microglia communication is a bi-direction way. Combining with the fact that exosomes derived from microglia play a role in tau and Aβ transmission in the brain as a delivery cargo, conversely, neuronal exosomes have similar effects on microglia. Wang et al. have demonstrated in their researches that microglia themselves could uptake neuronal exosomes, which containing intact and hypophosphorylated tau or Aβ, allowing them to play a scavenging role (Prada et al., 2013; Wang et al., 2017). Besides, Chang et al. (2013) have also revealed that intracerebroventricularly injection of exosomes derived from N2a cells in APP overexpression model of AD reduced Aβ levels, amyloid peptides deposition, as well as the Aβ-mediated synaptotoxicity in the hippocampus. This beneficial effect is further proved to be correlated with the internalization of glycosphingolipid-enriched N2a exosomes into microglia, as N2a cell-derived exosomes can act on extracellular Aβ, drive conformational changes of Aβ to form nontoxic amyloid fibrils, and promote uptake of Aβ by microglia (Yuyama et al., 2014). The internalized Aβ along with exosomes themselves were further transported to lysosomes for degradation (Brites and Vaz, 2014). These findings illustrate a coordinated mechanism by which neurons and neighboring microglia work together to clear Aβ peptides *via* exosomes. However, considering that not all evidence is supportive of the protective role of microglia in taking up neuronal exosomes, further researches are supposed to gain more insights into the protective roles as well as the pathogenic effects of microglial exosomes in neurodegenerative diseases.

## Microglia and Their Exosomes in PD

Parkinson’s disease is the most prevalent movement disorder, affecting about 1% of the population aged over 60 years old in the world (Samii et al., 2004). Along with physical traits like rigidity, bradykinesia, rest tremor, and postural instability, pathologically PD is characterized by greatly diminished dopaminergic neurons in the substantia nigra as well as the presence of Lewy bodies in the remaining neurons. α-synuclein (α-syn) is the major component of Lewy bodies and is prone to misfold to form fibrillar aggregation and propagate by shipping toxic agents between cell to cell (Ghidoni et al., 2008). Recent studies have focused on the exosomal transmission of α-syn, and α-syn in CNS-derived exosomes have been detected to be elevated in the plasma of PD patients (Shi et al., 2014; Kadam and Chuan, 2016).

Oligomeric α-syn can serve as the toxic seed for α-syn propagation. Recent evidence has indicated increased toxicity of exosomal α-syn oligomers, and they can be preferentially internalized by neurons (Danzer et al., 2012). Besides, it has been demonstrated the lipid component of the exosome can accelerate the deposition of α-syn (Grey et al., 2015). Evidence is also available showing that α-syn can induce an increased secretion of α-syn containing exosome by microglia (Chang et al., 2013). Our recent study systematically investigated the role of exosomes in mediating α-syn transmission, mainly focusing on the transmission from microglia to neurons (Guo et al., 2020). We found that when treated with human α-syn pre-formed fibrils (PFF), microglia were activated and secreted more exosomes. These exosomes contain α-syn monomer and oligomer, but no fibril and phosphorylated α-syn, and are fully capable of being uptaken by neurons and inducing α-syn protein aggregation in the recipient neurons. The function of microglia-derived exosomes in mediating α-syn transmission was further verified by inhibiting exosome secretion by GW4869, which leads to reduced neuronal uptake and reduced α-syn aggregation in neurons. In addition, stereotaxic injection of exosomes isolated from PFF treated microglia into the mouse striatum induces phosphorylated α-syn aggregation in different brain regions, such as cortex, hippocampus, cerebellum, and substantia nigra, confirming the ability of α-syn carrying exosomes from microglia in α-syn spreading *in vivo*. These animals exhibit dopaminergic neuron loss in the nigrostriatal pathway, which occurred in a time-dependent manner, accompanied by movement disorder at 180 days post-injection. These findings demonstrate that microglial exosomes can serve as potential intervention targets to limit α-syn transmission between cells in the brain.

Exosomes mediated α-syn protein transmission is closely related to autophagy, as the endosome pathway and autophagy pathway interact with each other in the clearance of α-syn protein (Alvarez-Erviti et al., 2011; Poehler et al., 2014). Autophagosomes have been proved to affect exosomes because autophagosomes can fuse with MVBs and then promotes exosomes secretion (Minakaki et al., 2018). Lysosome dysfunction inhibits autophagy but promotes the exosome-mediated protein spreading, for example, prions and α-syn (Vella et al., 2007; Fan et al., 2019). α-syn can impair autophagy in microglia, which is also confirmed in our recent work reporting that PFF treatment impaired autophagy flux in microglia (Fan et al., 2019; Guo et al., 2020). In PFF activated microglia, high expressed Peli-1 (pellino-1), an E3 ubiquitin ligase, induces lysosome breakdown and autophagy inhibition through degrading LAMP2, which promotes the secretion of α-syn carrying exosomes. Inhibiting Peli-1 in microglia restores lysosome function and reduces α-syn transmission *via* exosomes (Guo et al., 2020). We also found that inhibition of dynamin-related protein (Drp-1), a protein known for its function in mitochondrial fission, could enhance autophagy flux and inhibit α-syn carrying exosomes release, thus reducing microglia to neuron transmission and α-syn aggregation in neurons (Fan et al., 2019). These works support that targeting microglial exosomes is a potential therapeutic application in regulating the α-syn pathology in PD.

Although PD-associated inflammation induced by α-syn is well established, the effects and mechanisms of exosomes in regulating or propagating inflammation between microglia and neurons are still not clear. Little data is available regarding the exosome related inflammatory response. Previous studies found that MHC II and membrane TNF (mTNF-α) are enriched in exosomes from α-syn treated BV-2 cells, while the levels of TNF-converting enzyme (TACE), the transformation from mTNF-α to secretory TNF-α (sTNF-α), was unchanged, therefore, leading to enhanced cytotoxicity and eventually, increased neuronal apoptosis (Chang et al., 2013). Our recent work showed that when combined with microglial proinflammatory cytokines, oligomer carrying exosomes derived from primary microglia further increased α-syn protein aggregation in neurons, indicating the interaction of cytokines and exosomes (Guo et al., 2020). How microglial exosomes interact with inflammatory factors in α-syn pathology need to be further elucidated.

Studies showed that exosomes exist in CSF of PD patients, and these exosomes contain α-syn (Stuendl et al., 2016; Ngolab et al., 2017; Minakaki et al., 2018; Guo et al., 2020). In addition, it was reported that total exosomes from CSF of PD patients could induce α-syn aggregation, showing infectious abilities. For example, injection of CSF-derived exosomes from patients with dementia with Lewy bodies (DLB) and PD can induce the soluble α-syn accumulation to oligomer in recipient neurons as well as the mouse brain, located surrounding the injected site (Stuendl et al., 2016; Ngolab et al., 2017; Minakaki et al., 2018). However, the cellular sources of CSF-derived exosomes were not identified and mixed exosomes were used in these studies. Total exosomes in CSF including those from neurons, glia, and endothelial, and different cell-derived exosomes have different functions and properties (Budnik et al., 2016). In our study we used CD11b+ coated beads to purify microglia/macrophage-derived exosomes (CD11b+) and found that CD11b+ exosomes were present in the CSF of PD patients, comprising about 4–12% of the total exosomes. When cultured with neurons, these CD11b+ exosomes are able to infect neurons, functioning as seeds to induce the oligomerization of endogenous soluble α-syn inside neurons (Guo et al., 2020). This observation is particularly interesting when considering the possibility that peripherally activated macrophages and exosomes can invade the brain and thus massively impact microglia phenotype and CNS inflammation (Hawkes and McLaurin, 2009; Kierdorf et al., 2019). Macrophages exist at the CNS interfaces like dural, leptomeningeal, choroid plexus, and perivascular but not in the parenchyma in normal condition, while these macrophages can participate in pro-inflammatory response and disease-related α-syn spreading during PD (Thomas et al., 2007; Kovacs et al., 2014). Besides, exosomes from activated macrophages can invade the brain and modulate the M1–M2 phenotype of microglia and thus regulating inflammation response in the ischemic stroke model (Zheng et al., 2019). However, the function of macrophages and their exosomes in neurodegenerative disease still needs to be further investigated.

As aging is the most significant risk factor for PD, which is supported by both epidemiological studies and animal models of PD (Phinney et al., 2006). Consistent with this, Bliederhaeuser et al. (2016) have revealed in their study that microglia isolated from adult mice, rather than microglia from young mice, displayed deficits in the phagocytosis of both free and exosome-associated α-syn oligomers, combined with enhanced secretion of TNFα. Besides, by comparing monocytes from the elderly donors to young people, they also demonstrated impaired phagocytosis of α-syn in the periphery (Bliederhaeuser et al., 2016). Thus there is also a need to investigate the difference between young and aged microglia in PD, especially the ability in exosome release, misfolded protein phagocytosis, inflammatory response, cytokines release, and communication with neuronal cells. Fully understand this difference may help to reveal the more accurate roles of microglia and their exosomes in the pathology of PD.

## Microglia and Their Exosomes in ALS

Amyotrophic lateral sclerosis, characterized by progressive muscular paralysis, is a fatal neuromuscular degenerative disorder. Studies have found that in familial ALS (fALS), exosomes can mediate the spreading of misfolded or mutant copper-zinc superoxide dismutase one (mSOD1) between cells (Silverman et al., 2016). N9 microglial cells demonstrated a significant reduction in phagocytic ability when incubated with mSOD1 carrying exosomes. Besides, it induced M1 polarization in the early stage and mixed M1 and M2 subpopulations in the late stage in those exosome-treated N9 cells (Pinto et al., 2017). Nevertheless, TDP-43, the pathological hallmark of ALS, was detected in secreted exosomes from neuronal cells like N2a cells and primary neurons, yet not from glial cells like astrocytes or microglia (Iguchi et al., 2016). Moreover, Zondler et al. (2017) have demonstrated that exosomal TDP-43 can lead to the activation of peripheral monocyte and impaired the monocytic pro-inflammatory cytokine secretion. Till now, not much evidence of microglial exosomes in ALS has been reported, leaving a lot of unknowns.

## Conclusion and Future Directions

Exosomes are promising therapeutic applications that offer a wide range of opportunities for neurodegenerative diseases. Exosomes can be exploited as unmodified exosomes as well as being specifically loaded with cargos such as drugs, proteins, or nuclei, making them promising therapeutic carriers (Rufino-Ramos et al., 2017). Modification of microglial exosomes can be achieved by reforming the exosome-derived cells or through direct loaded. Direct loading of exosomes is achieved after exosomes are isolated. Therapeutic molecules such as RNAs, drugs, proteins, can be loaded into exosomes through passive incubation, as well as active methods, for example, freeze-thaw cycles, electroporation, sonication, and saponin treatment (Kosaka et al., 2010; Zhuang et al., 2011; Tang et al., 2012; Ingato et al., 2016). Manipulating of microglia can be achieved by the incubation with stimulating molecules to induce certain cell type, like M1 or M2 phenotype, as well as by transduction or transfection with expression vectors which lead to the secretion of exosomes containing specific molecules such as viral proteins, receptors, nucleic acids or RNA interacting proteins.

The therapeutic use of microglial EVs has been described in multiple neurodegenerative disorders. For instance, a reduction in EV release could be attributed to Aβ aggregation and AD pathogenesis exacerbation. In line with this, it has been shown that uptake of neuroblastoma-derived EVs in the brain could mediate Aβ clearance in the AD mouse model, as EVs functioned in transporting Aβ peptides and presenting them to microglia for degradation (Yuyama et al., 2014, 2015). For now, little is known about using microglial exosomes themselves or modified exosomes as therapeutic applications. Given that microglia can polarize to multiple phenotypes, for example, satellite microglia, KSPG-microglia, CD11c-microglia, Hox8b-microglia, and dark microglia, and their protein expression varied significantly (Stratoulias et al., 2019). Hence, the exosome from different subtypes might have distinct functions and contents, which give directions to future researches.

Activation of microglia has been indicated in neurodegenerative disease and activated microglia play an important role in both direct and indirect ways. The uptake of microglial exosomes by other cells and microglia phagocytosis exosomes from other cells mediate the indirect mechanisms, functioning in a cell-to-cell communication manner. This communication is important in the progression of the disease, whereas limited knowledge has been obtained from current studies. More research is needed to understand the mechanisms of microglial exosomes in neurodegenerative disease, and exploring the potential clinical usage of them in the future.

## Author Contributions

QD, MC, MG, and YH researched data for the review, wrote and revised the manuscript, contributed substantially to discussions of its content, and undertook review and editing of the manuscript before submission. HL, YF, and YM helped with the revision and figure preparation. All the authors read, polished, amended, and approved the final manuscript. All authors contributed to the article and approved the submitted version.

## Conflict of Interest

The authors declare that the research was conducted in the absence of any commercial or financial relationships that could be construed as a potential conflict of interest.

## Abbreviations

AD: Alzheimer’s disease
PD: Parkinson’s disease
ALS: amyotrophic lateral sclerosis
IFN- γ: interferon-gamma
DCs: dendritic cells
Drp-1: dynamin-related protein
DLB: dementia with Lewy bodies
mSOD1: mutant copper-zinc superoxide dismutase 1
MHC: major histocompatibility complex
CSF: cerebrospinal fluids
TNF: tumor necrosis factors.

## References

[B1] Alvarez-Erviti L. Seow Y. Schapira A. H. Gardiner C. Sargent I. L. Wood M. J. (2011). Lysosomal dysfunction increases exosome-mediated alpha-synuclein release and transmission. *Neurobiol. Dis.* 42 360–367. 10.1016/j.nbd.2011.01.029 21303699 PMC3107939

[B2] Asai H. Ikezu S. Tsunoda S. Medalla M. Luebke J. Haydar T. (2015). Depletion of microglia and inhibition of exosome synthesis halt tau propagation. *Nat. Neurosci.* 18 1584–1593. 10.1038/nn.4132 26436904 PMC4694577

[B3] Baietti M. F. Zhang Z. Mortier E. Melchior A. Degeest G. Geeraerts A. (2012). Syndecan-syntenin-ALIX regulates the biogenesis of exosomes. *Nat. Cell Biol.* 14 677–685. 10.1038/ncb2502 22660413

[B4] Basso M. Bonetto V. (2016). Extracellular vesicles and a novel form of communication in the brain. *Front. Neurosci.* 10:127. 10.3389/fnins.2016.00127 27065789 PMC4814526

[B5] Binotti B. Jahn R. Chua J. J. (2016). Functions of rab proteins at presynaptic sites. *Cells* 5:7. 10.3390/cells5010007 26861397 PMC4810092

[B6] Bliederhaeuser C. Grozdanov V. Speidel A. Zondler L. Ruf W. P. Bayer H. (2016). Age-dependent defects of alpha-synuclein oligomer uptake in microglia and monocytes. *Acta Neuropathol.* 131 379–391. 10.1007/s00401-015-1504-2 26576561

[B7] Brites D. Vaz A. R. (2014). Microglia centered pathogenesis in ALS: insights in cell interconnectivity. *Front. Cell. Neurosci.* 8:117. 10.3389/fncel.2014.00117 24904276 PMC4033073

[B8] Budnik V. Ruiz-Canada C. Wendler F. (2016). Extracellular vesicles round off communication in the nervous system. *Nat. Rev. Neurosci.* 17 160–172. 10.1038/nrn.2015.29 26891626 PMC4989863

[B9] Chang C. Lang H. Geng N. Wang J. Li N. Wang X. (2013). Exosomes of BV-2 cells induced by alpha-synuclein: important mediator of neurodegeneration in PD. *Neurosci. Lett.* 548 190–195. 10.1016/j.neulet.2013.06.009 23792198

[B10] Chevallier J. Koop C. Srivastava A. Petrie R. J. Lamarche-Vane N. Presley J. F. (2009). Rab35 regulates neurite outgrowth and cell shape. *FEBS Lett.* 583 1096–1101. 10.1016/j.febslet.2009.03.012 19289122

[B11] Danzer K. M. Kranich L. R. Ruf W. P. Cagsal-Getkin O. Winslow A. R. Zhu L. (2012). Exosomal cell-to-cell transmission of alpha synuclein oligomers. *Mol. Neurodegener.* 7:42. 10.1186/1750-1326-7-42 22920859 PMC3483256

[B12] Deczkowska A. Keren-Shaul H. Weiner A. Colonna M. Schwartz M. Amit I. (2018). Disease-associated microglia: a universal immune sensor of neurodegeneration. *Cell* 173 1073–1081. 10.1016/j.cell.2018.05.003 29775591

[B13] Dinkins M. B. Dasgupta S. Wang G. Zhu G. Bieberich E. (2014). Exosome reduction in vivo is associated with lower amyloid plaque load in the 5XFAD mouse model of Alzheimer’s disease. *Neurobiol. Aging* 35 1792–1800. 10.1016/j.neurobiolaging.2014.02.012 24650793 PMC4035236

[B14] Dinkins M. B. Dasgupta S. Wang G. Zhu G. He Q. Kong J. N. (2015). The 5XFAD mouse model of Alzheimer’s disease exhibits an age-dependent increase in anti-ceramide IgG and exogenous administration of ceramide further increases anti-ceramide titers and amyloid plaque burden. *J. Alzheimers Dis.* 46 55–61. 10.3233/jad-150088 25720409 PMC4593501

[B15] Fan R. Z. Guo M. Luo S. Cui M. Tieu K. (2019). Exosome release and neuropathology induced by alpha-synuclein: new insights into protective mechanisms of Drp1 inhibition. *Acta Neuropathol. Commun.* 7:184. 10.1186/s40478-019-0821-4 PMC6862865 31744532

[B16] Fernandes A. Ribeiro A. R. Monteiro M. Garcia G. Vaz A. R. Brites D. (2018). Secretome from SH-SY5Y APPSwe cells trigger time-dependent CHME3 microglia activation phenotypes, ultimately leading to miR-21 exosome shuttling. *Biochimie* 155 67–82. 10.1016/j.biochi.2018.05.015 29857185

[B17] Fruhbeis C. Frohlich D. Kuo W. P. Kramer-Albers E. M. (2013). Extracellular vesicles as mediators of neuron-glia communication. *Front. Cell. Neurosci.* 7:182. 10.3389/fncel.2013.00182 24194697 PMC3812991

[B18] Gemma C. Bachstetter A. D. (2013). The role of microglia in adult hippocampal neurogenesis. *Front. Cell. Neurosci.* 7:229. 10.3389/fncel.2013.00229 24319411 PMC3837350

[B19] Ghidoni R. Benussi L. Binetti G. (2008). Exosomes: the Trojan horses of neurodegeneration. *Med. Hypotheses* 70 1226–1227. 10.1016/j.mehy.2007.12.003 18226468

[B20] Glebov K. Lochner M. Jabs R. Lau T. Merkel O. Schloss P. (2015). Serotonin stimulates secretion of exosomes from microglia cells. *Glia* 63 626–634. 10.1002/glia.22772 25451814

[B21] Gouwens L. K. Ismail M. S. Rogers V. A. Zeller N. T. Garrad E. C. Amtashar F. S. (2018). Abeta42 protofibrils interact with and are trafficked through microglial-derived microvesicles. *ACS Chem. Neurosci.* 9 1416–1425. 10.1021/acschemneuro.8b00029 29543435

[B22] Grey M. Dunning C. J. Gaspar R. Grey C. Brundin P. Sparr E. (2015). Acceleration of alpha-synuclein aggregation by exosomes. *J. Biol. Chem.* 290 2969–2982. 10.1074/jbc.m114.585703 25425650 PMC4317028

[B23] Gross J. C. Chaudhary V. Bartscherer K. Boutros M. (2012). Active Wnt proteins are secreted on exosomes. *Nat. Cell Biol.* 14 1036–1045. 10.1038/ncb2574 22983114

[B24] Gu B. J. Saunders B. M. Petrou S. Wiley J. S. (2011). P2X(7) is a scavenger receptor for apoptotic cells in the absence of its ligand, extracellular ATP. *J. Immunol.* 187 2365–2375. 10.4049/jimmunol.1101178 21821797

[B25] Guo M. Wang J. Zhao Y. Feng Y. Han S. Dong Q. (2020). Microglial exosomes facilitate alpha-synuclein transmission in Parkinson’s disease. *Brain* 143 1476–1497. 10.1093/brain/awaa090 32355963 PMC7241957

[B26] Hawkes C. A. McLaurin J. (2009). Selective targeting of perivascular macrophages for clearance of beta-amyloid in cerebral amyloid angiopathy. *Proc. Natl. Acad. Sci. U.S.A.* 106 1261–1266. 10.1073/pnas.0805453106 19164591 PMC2633563

[B27] Hooper C. Sainz-Fuertes R. Lynham S. Hye A. Killick R. Warley A. (2012). Wnt3a induces exosome secretion from primary cultured rat microglia. *BMC Neurosci.* 13:144. 10.1186/1471-2202-13-144 23173708 PMC3541220

[B28] Iguchi Y. Eid L. Parent M. Soucy G. Bareil C. Riku Y. (2016). Exosome secretion is a key pathway for clearance of pathological TDP-43. *Brain* 139 3187–3201. 10.1093/brain/aww237 27679482 PMC5840881

[B29] Ingato D. Lee J. U. Sim S. J. Kwon Y. J. (2016). Good things come in small packages: overcoming challenges to harness extracellular vesicles for therapeutic delivery. *J. Control. Release* 241 174–185. 10.1016/j.jconrel.2016.09.016 27667180

[B30] Joshi P. Turola E. Ruiz A. Bergami A. Libera D. D. Benussi L. (2014). Microglia convert aggregated amyloid-beta into neurotoxic forms through the shedding of microvesicles. *Cell Death Differ.* 21 582–593. 10.1038/cdd.2013.180 24336048 PMC3950321

[B31] Kadam P. D. Chuan H. H. (2016). Rectocutaneous fistula with transmigration of the suture: a rare delayed complication of vault fixation with the sacrospinous ligament. *Int. Urogynecol. J.* 27:505. 10.1007/s00192-016-2952-5 26318612

[B32] Keren-Shaul H. Spinrad A. Weiner A. Matcovitch-Natan O. Dvir-Szternfeld R. Ulland T. K. (2017). A unique microglia type associated with restricting development of Alzheimer’s disease. *Cell* 169 1276–1290.e17. 28602351 10.1016/j.cell.2017.05.018

[B33] Kierdorf K. Masuda T. Jordao M. J. C. Prinz M. (2019). Macrophages at CNS interfaces: ontogeny and function in health and disease. *Nat. Rev. Neurosci.* 20 547–562. 10.1038/s41583-019-0201-x 31358892

[B34] Kosaka N. Iguchi H. Yoshioka Y. Takeshita F. Matsuki Y. Ochiya T. (2010). Secretory mechanisms and intercellular transfer of microRNAs in living cells. *J. Biol. Chem.* 285 17442–17452. 10.1074/jbc.m110.107821 20353945 PMC2878508

[B35] Kovacs G. G. Breydo L. Green R. Kis V. Puska G. Lorincz P. (2014). Intracellular processing of disease-associated α-synuclein in the human brain suggests prion-like cell-to-cell spread. *Neurobiol Dis.* 69, 76–92. 10.1016/j.nbd.2014.05.020 24878508

[B36] Lambert J. C. Ibrahim-Verbaas C. A. Harold D. Naj A. C. Sims R. Bellenguez C. (2013). Meta-analysis of 74,046 individuals identifies 11 new susceptibility loci for Alzheimer’s disease. *Nat. Genet.* 45 1452–1458. 24162737 10.1038/ng.2802 PMC3896259

[B37] Minakaki G. Menges S. Kittel A. Emmanouilidou E. Schaeffner I. Barkovits K. (2018). Autophagy inhibition promotes SNCA/alpha-synuclein release and transfer via extracellular vesicles with a hybrid autophagosome-exosome-like phenotype. *Autophagy* 14 98–119. 10.1080/15548627.2017.1395992 29198173 PMC5846507

[B38] Ngolab J. Trinh I. Rockenstein E. Mante M. Florio J. Trejo M. (2017). Brain-derived exosomes from dementia with Lewy bodies propagate alpha-synuclein pathology. *Acta Neuropathol. Commun.* 5:46. 10.1186/s40478-017-0445-5 PMC5466770 28599681

[B39] Olah M. Patrick E. A.-O. Villani A. C. Xu J. A.-O. White C. C. Ryan K. J. (2018). A transcriptomic atlas of aged human microglia. *Nat. Commun.* 9:539. 10.1038/s41467-018-02926-5 PMC5803269 29416036

[B40] Peferoen L. Kipp M. van der Valk P. van Noort J. M. Amor S. (2014). Oligodendrocyte-microglia cross-talk in the central nervous system. *Immunology* 141 302–313. 10.1111/imm.12163 23981039 PMC3930369

[B41] Perez-Gonzalez R. Gauthier S. A. Kumar A. Levy E. (2012). The exosome secretory pathway transports amyloid precursor protein carboxyl-terminal fragments from the cell into the brain extracellular space. *J. Biol. Chem.* 287 43108–43115. 10.1074/jbc.m112.404467 23129776 PMC3522305

[B42] Phinney A. L. Andringa G. Bol J. G. J. M. Wolters E. C. Muiswinkel F. L. V. Dam A.-M. W. V. (2006). Enhanced sensitivity of dopaminergic neurons to rotenone-induced toxicity with aging. *Parkinsonism Relat. Disord.* 12 228–238. 10.1016/j.parkreldis.2005.12.002 16488175

[B43] Pinto S. Cunha C. Barbosa M. Vaz A. R. Brites D. (2017). Exosomes from NSC-34 cells transfected with hSOD1-G93A are enriched in miR-124 and drive alterations in microglia phenotype. *Front. Neurosci.* 11:273. 10.3389/fnins.2017.00273 28567000 PMC5434170

[B44] Poehler A. M. Xiang W. Spitzer P. May V. E. Meixner H. Rockenstein E. (2014). Autophagy modulates SNCA/alpha-synuclein release, thereby generating a hostile microenvironment. *Autophagy* 10 2171–2192. 10.4161/auto.36436 25484190 PMC4502760

[B45] Potolicchio I. Carven G. J. Xu X. Stipp C. Riese R. J. Stern L. J. (2005). Proteomic analysis of microglia-derived exosomes: metabolic role of the aminopeptidase CD13 in neuropeptide catabolism. *J. Immunol.* 175 2237–2243. 10.4049/jimmunol.175.4.2237 16081791

[B46] Prada I. Furlan R. Matteoli M. Verderio C. (2013). Classical and unconventional pathways of vesicular release in microglia. *Glia* 61 1003–1017. 10.1002/glia.22497 23625857

[B47] Ratajczak J. Wysoczynski M. Hayek F. Janowska-Wieczorek A. Ratajczak M. Z. (2006). Membrane-derived microvesicles: important and underappreciated mediators of cell-to-cell communication. *Leukemia* 20 1487–1495. 10.1038/sj.leu.2404296 16791265

[B48] Rufino-Ramos D. Albuquerque P. R. Carmona V. Perfeito R. Nobre R. J. Pereira de Almeida L. (2017). Extracellular vesicles: novel promising delivery systems for therapy of brain diseases. *J. Control. Release* 262 247–258. 10.1016/j.jconrel.2017.07.001 28687495

[B49] Samii A. Nutt J. G. Ransom B. R. (2004). Parkinson’s disease. *Lancet* 363 1783–1793. 15172778 10.1016/S0140-6736(04)16305-8

[B50] Sardar Sinha M. Ansell-Schultz A. Civitelli L. Hildesjo C. Larsson M. Lannfelt L. (2018). Alzheimer’s disease pathology propagation by exosomes containing toxic amyloid-beta oligomers. *Acta Neuropathol.* 136 41–56. 10.1007/s00401-018-1868-1 29934873 PMC6015111

[B51] Shi M. Liu C. Cook T. J. Bullock K. M. Zhao Y. Ginghina C. (2014). Plasma exosomal alpha-synuclein is likely CNS-derived and increased in Parkinson’s disease. *Acta Neuropathol.* 128 639–650. 10.1007/s00401-014-1314-y 24997849 PMC4201967

[B52] Shifrin D. A. Jr. Demory Beckler M. Coffey R. J. Tyska M. J. (2013). Extracellular vesicles: communication, coercion, and conditioning. *Mol. Biol. Cell* 24 1253–1259. 10.1091/mbc.e12-08-0572 23630232 PMC3639038

[B53] Silverman J. M. Fernando S. M. Grad L. I. Hill A. F. Turner B. J. Yerbury J. J. (2016). Disease mechanisms in ALS: misfolded SOD1 transferred through exosome-dependent and exosome-independent pathways. *Cell. Mol. Neurobiol.* 36 377–381. 10.1007/s10571-015-0294-3 26908139 PMC11482315

[B54] Stratoulias V. Venero J. L. Tremblay M. E. Joseph B. (2019). Microglial subtypes: diversity within the microglial community. *EMBO J.* 38:e101997. 10.15252/embj.2019101997 PMC6717890 31373067

[B55] Stuendl A. Kunadt M. Kruse N. Bartels C. Moebius W. Danzer K. M. (2016). Induction of alpha-synuclein aggregate formation by CSF exosomes from patients with Parkinson’s disease and dementia with Lewy bodies. *Brain* 139 481–494. 10.1093/brain/awv346 26647156 PMC4805087

[B56] Tang K. Zhang Y. Zhang H. Xu P. Liu J. Ma J. (2012). Delivery of chemotherapeutic drugs in tumour cell-derived microparticles. *Nat. Commun.* 3:1282. 10.1038/ncomms2282 23250412

[B57] Thomas M. P. Chartrand K. Reynolds A. Vitvitsky V. Banerjee R. Gendelman H. E. (2007). Ion channel blockade attenuates aggregated alpha synuclein induction of microglial reactive oxygen species: relevance for the pathogenesis of Parkinson’s disease. *J. Neurochem.* 100, 503–519. 10.1111/j.1471-4159.2006.04315.x 17241161

[B58] Turola E. Furlan R. Bianco F. Matteoli M. Verderio C. (2012). Microglial microvesicle secretion and intercellular signaling. *Front. Physiol.* 3:149. 10.3389/fphys.2012.00149 22661954 PMC3357554

[B59] Valdinocci D. Radford R. A. Siow S. M. Chung R. S. Pountney D. L. (2017). Potential modes of intercellular alpha-synuclein transmission. *Int. J. Mol. Sci.* 18:469. 10.3390/ijms18020469 28241427 PMC5344001

[B60] Vella L. J. Sharples R. A. Lawson V. A. Masters C. L. Cappai R. Hill A. F. (2007). Packaging of prions into exosomes is associated with a novel pathway of PrP processing. *J. Pathol.* 211 582–590. 10.1002/path.2145 17334982

[B61] Verderio C. Muzio L. Turola E. Bergami A. Novellino L. Ruffini F. (2012). Myeloid microvesicles are a marker and therapeutic target for neuroinflammation. *Ann. Neurol.* 72 610–624. 10.1002/ana.23627 23109155

[B62] Wang Y. Balaji V. Kaniyappan S. Kruger L. Irsen S. Tepper K. (2017). The release and trans-synaptic transmission of Tau via exosomes. *Mol. Neurodegener.* 12:5. 10.1186/s13024-016-0143-y PMC5237256 28086931

[B63] Yuyama K. Igarashi Y. (2017). Exosomes as carriers of Alzheimer’s amyloid-ss. *Front. Neurosci.* 11:229. 10.3389/fnins.2017.00229 28487629 PMC5403946

[B64] Yuyama K. Sun H. Mitsutake S. Igarashi Y. (2012). Sphingolipid-modulated exosome secretion promotes clearance of amyloid-beta by microglia. *J. Biol. Chem.* 287 10977–10989. 10.1074/jbc.m111.324616 22303002 PMC3322859

[B65] Yuyama K. Sun H. Sakai S. Mitsutake S. Okada M. Tahara H. (2014). Decreased amyloid-beta pathologies by intracerebral loading of glycosphingolipid-enriched exosomes in Alzheimer model mice. *J. Biol. Chem.* 289 24488–24498. 10.1074/jbc.m114.577213 25037226 PMC4148874

[B66] Yuyama K. Sun H. Usuki S. Sakai S. Hanamatsu H. Mioka T. (2015). A potential function for neuronal exosomes: sequestering intracerebral amyloid-beta peptide. *FEBS Lett.* 589 84–88. 10.1016/j.febslet.2014.11.027 25436414

[B67] Zheng Y. He R. Wang P. Shi Y. Zhao L. Liang J. (2019). Exosomes from LPS-stimulated macrophages induce neuroprotection and functional improvement after ischemic stroke by modulating microglial polarization. *Biomater. Sci.* 7, 2037–2049. 10.1039/C8BM01449C 30843911

[B68] Zhuang X. Xiang X. Grizzle W. Sun D. Zhang S. Axtell R. C. (2011). Treatment of brain inflammatory diseases by delivering exosome encapsulated anti-inflammatory drugs from the nasal region to the brain. *Mol. Ther.* 19 1769–1779. 10.1038/mt.2011.164 21915101 PMC3188748

[B69] Zondler L. Feiler M. S. Freischmidt A. Ruf W. P. Ludolph A. C. Danzer K. M. (2017). Impaired activation of ALS monocytes by exosomes. *Immunol. Cell Biol.* 95 207–214. 10.1038/icb.2016.89 27616750

